# *Bacillus amyloliquefaciens* Ameliorates Dextran Sulfate Sodium-Induced Colitis by Improving Gut Microbial Dysbiosis in Mice Model

**DOI:** 10.3389/fmicb.2018.03260

**Published:** 2019-01-08

**Authors:** Guangtian Cao, Kangli Wang, Zhanming Li, Fei Tao, Yinglei Xu, Junhong Lan, Guangyong Chen, Caimei Yang

**Affiliations:** ^1^College of Standardization, China Jiliang University, Hangzhou, China; ^2^Key Laboratory of Applied Technology on Green-Eco-Healthy Animal Husbandry of Zhejiang Province, College of Animal Science and Technology, Zhejiang A and F University, Hangzhou, China; ^3^Department of Food Science, China Jiliang University, Hangzhou, China; ^4^Zhejiang Provincial Engineering Laboratory for Animal Health Inspection and Internet Technology, College of Animal Science and Technology, Zhejiang A and F University, Hangzhou, China

**Keywords:** *Bacillus amyloliquefaciens*, colitis, inflammation, intestinal morphology, microbiota profiling

## Abstract

Several *Bacillus* strains exert beneficial effects on the maintenance of intestinal homeostasis and host health. However, whether *Bacillus amyloliquefaciens* (BA) can improve gut microbial dysbiosis and ameliorate colitis is unknown. Therefore, we conducted the present study to investigate the effects of BA administration on intestinal morphology, inflammatory response, and colonic microbial composition in a mouse model of dextran sulfate sodium (DSS)-induced colitis. Results showed that BA administration significantly ameliorated body weight loss, decreased disease activity index, and improved colonic tissue morphology in DSS-treated mice. In addition, levels of immunoglobulins, as well as pro-inflammatory cytokines, were decreased after BA administration. Importantly, colonic microbiota profiling indicated a significant (*p* < 0.05) difference in beta-diversity between BA-administrated and DSS-treated mice, according to weighted principal coordinate analysis (PCoA) results. The relative abundance of the *Firmicutes* genus was increased, whereas that of *Bacteroidetes* was decreased by BA administration. Furthermore, phylogenetic investigation of communities by reconstruction of unobserved states (PICRUSt) analysis showed that the most significantly changed pathways between the four groups of mice were carbohydrate, lipid, and amino acid metabolism. In conclusion, our results showed that BA administration has beneficial effects on DSS-induced colitis, suggesting that this strategy might be useful for the treatment of dysbiosis during ulcerative colitis. Further, the changes in metabolism, especially amino acid metabolism, might contribute to the beneficial effects of BA on the amelioration of DSS-induced colitis.

## Introduction

Inflammatory bowel disease (IBD) is a chronic inflammatory disease that mostly occurs in the rectal and colonic mucosa and even deeper layers of the intestinal wall. Ulcerative colitis (UC) and Crohn's disease (CD) are the two main forms of IBD, and are characterized by clinical symptoms such as weight loss, diarrhea, and rectal bleeding. Although evidence suggested that mucosal edema and altered innate and adaptive immune responses contribute to the development of these diseases (Munyaka et al., 2016), the precise mechanism of the IBD pathogenesis is still unknown.

Beyond inherent basic nutrition, oral probiotics also exert many beneficial effects on health. Several species of *Bacillus* can function as antibiotic alternatives and growth promoters as they improve the digestibility and absorption of nutrients in the intestines of pigs and birds (Hong et al., 2005; Cao et al., 2018). Especially, emerging evidence has suggested that *Bacillus amyloliquefaciens* (BA) is beneficial for the amelioration of diarrhea and inflammation (Li et al., 2018). One study also reported the beneficial effects of BA on IBD as it ameliorated the body weight loss of dextran sulfate sodium salt (DSS)-induced colitis animals, in addition to reducing the protein and mRNA levels of pro-inflammatory cytokines in colonic tissues (Hairul Islam et al., 2011). However, they did not report any related mechanisms including whether BA supplementation affected the composition of the gut microbiota.

IBD is characterized as functional dysbiosis in the intestine including dysbiosis of microbiota. As the most widely used experimental model of UC, DSS-induced colitis is associated with alterations to the gut microbiota. Therefore, we conducted the present study to investigate the effects of *B. amyloliquefaciens* (CGMCC 9384) on DSS-induced colitis mice and to determine whether it has any effects on gut microbiota dysbiosis.

## Materials and Methods

### Animal Experiments

Twenty-four C57/BL6 male mice aged 9 weeks and weighing 21–23 g were purchased from SLAC Laboratory Animal Central (Changsha, China). All mice were individually maintained under standard conditions (lighting cycle, 12 h/d; temperature, 22 ± 2°C; relative humidity, 50 ± 5%). Animals were randomly assigned into four groups as follows: mice were untreated for 7 days and then orally gavaged PBS for 7 days (Control group); mice were supplemented with 3.5% DSS (w/v; molecular mass = 6,500–10,000 Da; Sigma-Aldrich, Shanghai, China) dissolved in fresh running water *ad libitum* for 7 days and then orally gavaged PBS for 7 days (DSS group); mice were supplemented with 3.5% DSS for 7 days and then orally gavaged *B. amyloliquefaciens* (1.0 × 10^8^ CFU/kg in 200 μL of PBS/mouse/day) for 7 days (BaL (low-dose) group); mice were supplemented with 3.5% DSS for 7 days and then orally gavaged with *B. amyloliquefaciens* (1.0 × 10^9^ CFU/kg in 200 μL of PBS/mouse/day) for 7 days (BaH (high-dose) group). The probiotic *B. amyloliquefaciens* (CGMCC9384) was provided by Zhejiang Huijia Biological Technology Ltd., Anji, China. According to our previous study, after fermentation (37°C, 48 h) and drying, the strain was granulated and used at a level of approximately 10^10^ CFU/g (Cao et al., 2018). Diet was supplied by Research Diets Inc (New Brunswick, NJ, USA). At the end of the experiment, blood was obtained from the retro-orbital sinus, centrifuged at 4°C at 800 *g* for 10 min for serum collection. Then mice were euthanized by cervical dislocation for colon and colonic digesta sample collection. Body weight, colonic length, rectal bleeding, and stool consistency were recorded and scored according to standard protocols (Zhang et al., 2015; Wang et al., 2017). The scoring system for disease activity index (DAI) is shown in Table 1. All procedures in the present study were approved by the Animal Welfare Committee of China Jiliang University and all procedures were carried out according to the rules established by the committee.

**Table 1:** Scoring system for the disease activity index.

**Score**	**Weight loss**	**Rectal bleeding**	**Stool consistency**
0	0	normal	normal
1	0.1~5%	small amount of blood	loose stool
2	5~10%	blood in stool regularly seen	mild diarrhea
3	>10%	blood in all stool	diarrhea

### Histological Analyses

Colonic samples were fixed with 4% formaldehyde overnight, and then embedded in paraffin and cut into 8 μm thick sections as previous study described (Zhou X. et al., 2017; Zhou et al., 2018b). Following this, the sections were stained with hematoxylin and eosin (HE). Additionally, colonic samples were fixed with 2.5% glutaraldehyde at 4°C and embedded in Epon-Araldite resin. The ultrathin sections were stained with uranyl acetate and lead citrate and observed under a Zeiss 902 transmission electron microscope.

### Assessment of Apoptosis

The TUNEL method was used to stain apoptotic cells using an *in situ* cell death detection kit (Roche, Shanghai, China). Nuclei was stained with DAPI mounting solution (Vector, Burlingame, CA, USA). Apoptotic cells were observed under a light microscope (Leica, Solms, Germany) and representative pictures were taken.

### Measurement of Immunoglobulins (Ig), Inflammatory Cytokines, Myeloperoxidase (MPO), and Eosinophil Peroxidase (EPO) Concentrations

Commercially available kits from Cusabio Biotech Co., Ltd. (Wuhan, Hubei, China) were used to the determination of serum concentrations of IgG, IgA, and IgM, as well as the concentrations of IL-1β, TNF-α, IL-6, EPO, and MPO in the colon, which were performed in accordance with manufacturer's instructions.

### 16S rRNA Gene Sequencing

DNA from the colonic contents was isolated using the MoBio Power soil DNA Isolation Kit (Mo Bio Laboratories, Carlsbad, CA, United States) according to the manufacturer's manual. The concentration and purity of DNA were tested by 1% agarose gel electrophoresis, and DNA was diluted with sterile water to 1 ng/μL, which was stored at −80°C until sequencing (Yin et al., 2017, 2018). The V4 region of the colonic bacterial 16S rRNA genes was amplified using the specific primers 515F (5′-GTGCCAGCMGCCGCGGTAA-3′) and 806R (5′-GGACTACVSGGGTATCTAAT-3′). Purified PCR amplicons with a bright main band of 300 bp were sequenced with the Illumina MiSeq platform at Novogene (Beijing, China). After the filtering of low-quality genes, the chimeras in raw reads were removed using Cutadapt software (V1.9.1). The sequence database was built by the Ion Plus Fragment Library Kit 48 rxns (Thermo Fisher Scientific, US), and Ion S5^TM^XLplatform was used for further sequencing. The operational taxonomic units (OTUs) with a similarity threshold of 97% were selected with the UPARSE software package (v7.0.1001).

### Western Blot Analysis

Western blot analysis was conducted as previously described (Zhou X. H. et al., 2017; Zhou et al., 2018a). Extracts from colonic tissue samples containing equal quantities of proteins (30 μg) were electrophoresed on a polyacrylamide gel. Next, the separated proteins were transferred onto a PVDF membrane. Firstly, the membrane was incubated overnight at 4°C with antibodies against AMP-activated protein kinase (AMPK, rabbit monoclonal, diluted at 1:1,000), general amino acid control non-derepressible 2 (GCN2, rabbit polyclonal, diluted at 1:1,000), and mammalian target of rapamycin complex 1 (mTORC1, rabbit monoclonal, diluted at 1:1,000) (Cell Signaling, Beverly, MA, United States). Then, second antibody (diluted at 1:5,000; Cell Signaling) was incubated with the blots used for the incubation at 4°C for 2 h. Protein bands were developed with EZ-ECL (Biological Industries, Cromwell, CT, United States) by the ChemiDoc MP system (BIO-RAD, Hercules, CA, USA). All protein measurements were normalized to GAPDH (1:1,000; Proteintech, Rosemont, IL, USA) and data are expressed relative to the values in control mice.

### Sequencing Data Analysis

Based on the results of all sample species annotations using Mothur software and the SILVA database (SSUrRNA), the top 10 phyla and the top 10 genera were analyzed (relative abundance over 1%). The alpha diversity (Shannon index) and the unweighted pair-group method with Arithmetic Means (UPGMA) clustering were investigated using QIIME software (version 1.7.0). The beta diversity was calculated from unweighted and weighted UniFrac distances, in which principal coordinate analysis (PCoA) and non-metric multidimensional scaling (NMDS) analysis were performed to analyze the colonic microbial community data using R software (Version 2.15.3). In addition, analysis of similarity was performed to test significant differences between sample groups based on unweighted UniFrac distance matrices. Finally, the taxonomic files were achieved from QIIME software, and Linear discriminant analysis with effect size (LEfSe) analysis and phylogenetic investigation of communities by reconstruction of unobserved states (PICRUSt) were performed online (http://huttenhower.sph.harvard.edu/galaxy/).

### Statistical Analysis

All statistical analyses were performed by one-way ANOVA, using the general linear model and the MIXED procedure (PROC MIXED) from SAS software version 9.2 (SAS Institute Inc., Cary, NC, United States). Data are presented as least square means plus pooled SEM. *P* < 0.05 was considered statistically significant and 0.05 < *P* < 0.1 was considered as a tendency.

## Results

### Effects of *B. amyloliquefaciens* on Colitis Signs

**Figure 1:**
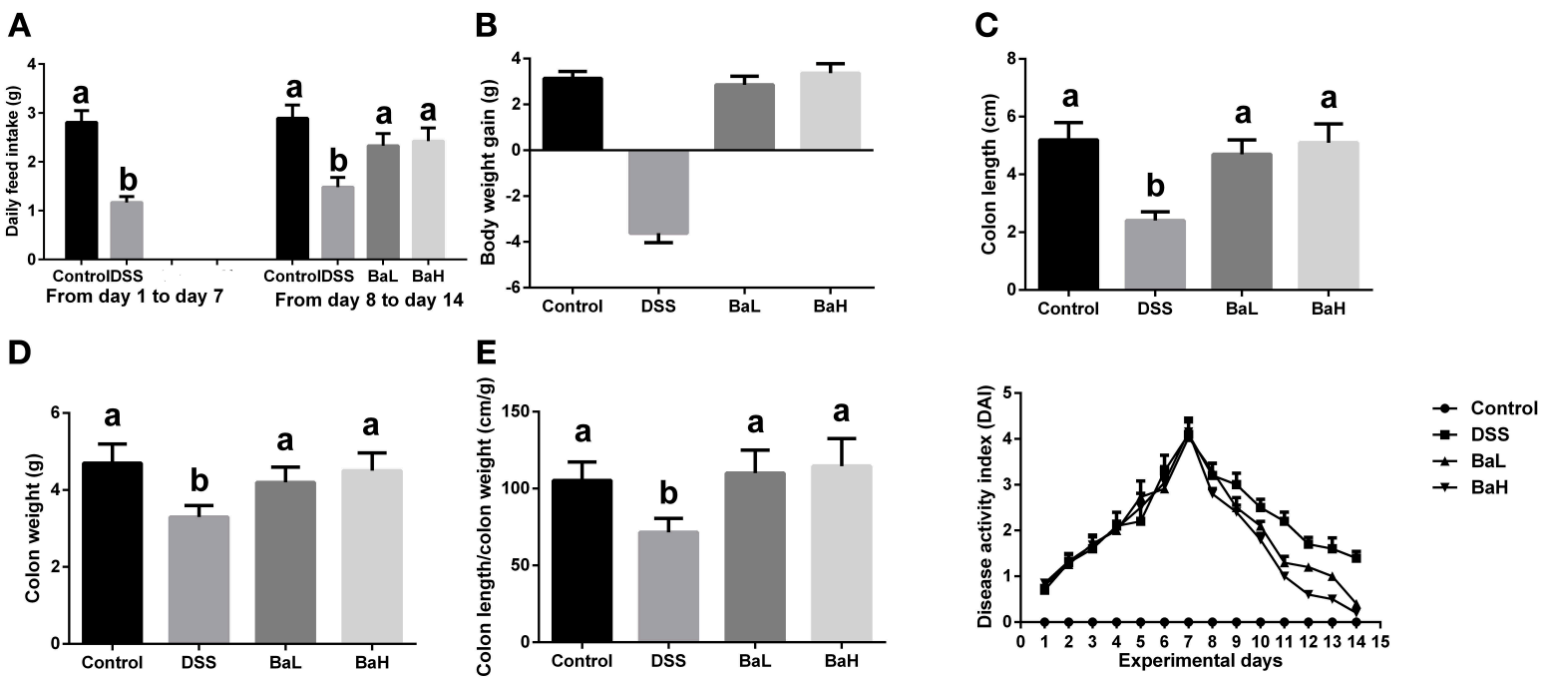
Effects of *B. amyloliquefaciens* on Colitis Signs. **(A)** Average daily feed intake. **(B)** Body weight gain. **(C)** Colon length. **(D)** Colon weight. **(E)** Colon length/colon weight ratio. **(F)** Disease activity index. Control, mice were untreated for 7 days and then orally gavaged PBS for 7 days; DSS, mice were supplemented with 3.5% DSS for 7 days and then orally gavaged PBS for 7 days; BaL, mice were supplemented with 3.5% DSS for 7 days and then orally gavaged *B. amyloliquefaciens* (Ba, 9.0 × 10^8^ CFU/kg in 200 μL of PBS/mouse/day) for 7 days; BaH, mice were supplemented with 3.5% DSS for 7 days and then orally gavaged *B. amyloliquefaciens* (Ba, 10.0 × 10^8^ CFU/kg in 200 μL of PBS/mouse/day).Values are expressed as mean ± SEM, *n* = 6. ^a,b^Means of the bars with different letters were significantly different among groups (*P* < 0.05).

The effects of BA on colitis signs in mice are shown in Figure 1. On day 14, both low and high levels of BA supplementation reversed DSS-induced feed intake and weight loss (Figures 1A,B). BA-supplemented mice exhibited increased colon lengths, colon weights, and colon length/colon weight ratios compared to DSS-treated mice (Figures 1C–E). Moreover, BA supplementation lowered the DAI from day 9 to day 14 (Figure 1F).

### Effects of *B. amyloliquefaciens* on DSS-induced Histopathological Damage and Apoptosis

**Figure 2:**
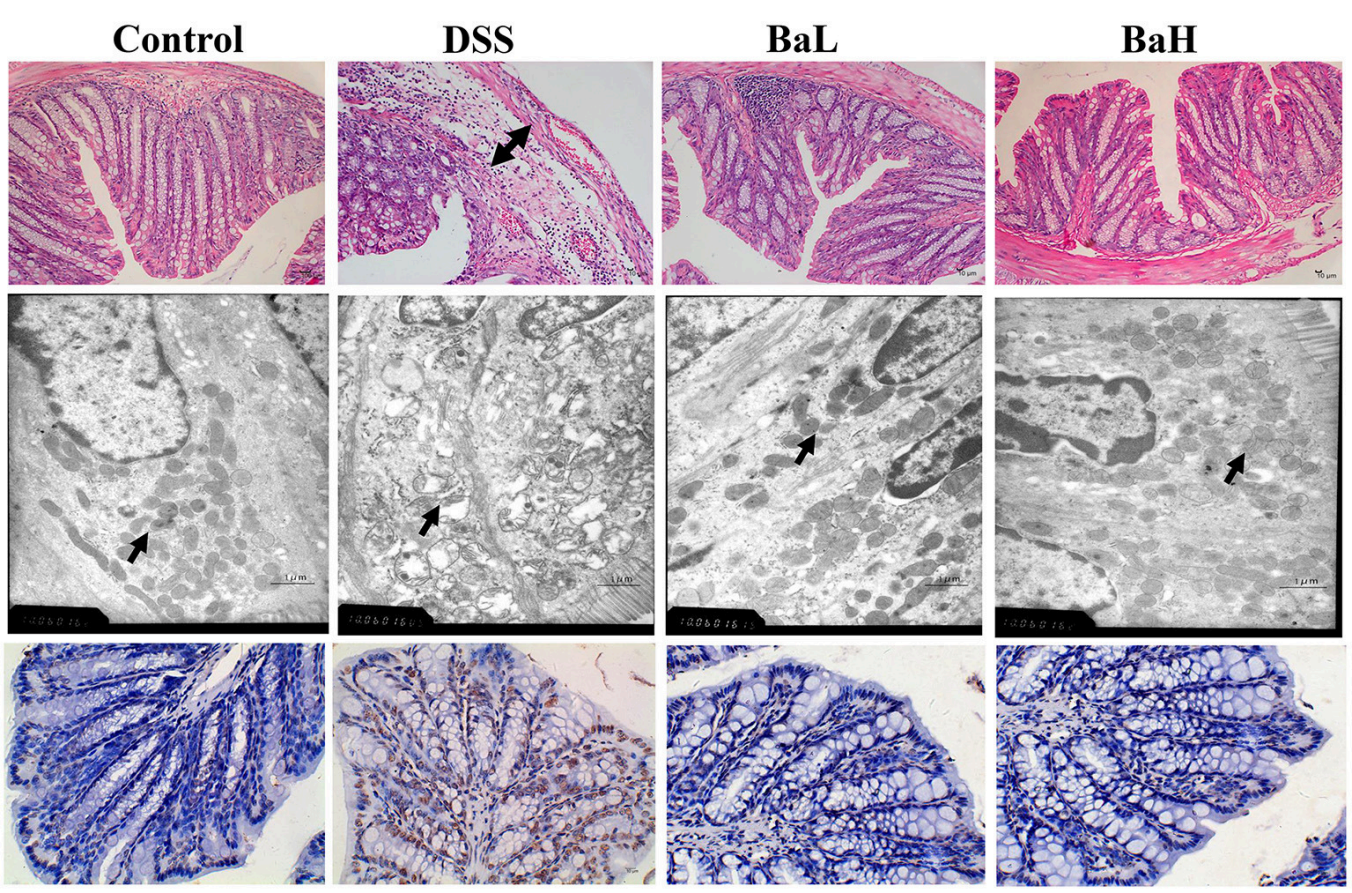
Effects of *B. amyloliquefaciens* on DSS-induced Histopathological Damage and Apoptosis. (Upper) HE staining of colon morphology (40×). Arrow, obvious edema. (Middle) ultrastructural observation of colon (transmission electron microscopy, 10,000×). Arrow, mitochondria. (Lower) TUNEL staining (yellow) for assessment of apoptosis (400×), nuclei were stained with DAPI (blue). Control, mice were untreated for 7 days and then orally gavaged PBS for 7 days; DSS, mice were supplemented with 3.5% DSS for 7 days and then orally gavaged PBS for 7 days; BaL, mice were supplemented with 3.5% DSS for 7 days and then orally gavaged *B. amyloliquefaciens* (Ba, 9.0 × 10^8^ CFU/kg in 200 μL of PBS/mouse/day) for 7 days; BaH, mice were supplemented with 3.5% DSS for 7 days and then orally gavaged *B. amyloliquefaciens* (Ba, 10.0 × 10^8^ CFU/kg in 200 μL of PBS/mouse/day).

The effects of BA supplementation on DSS-induced histopathological damage and apoptosis are shown in Figure 2. H&E staining results indicated pathological damage in the colon tissue of DSS-treated mice, such as obvious edema in the structure of the mucous layer, whereas BA-supplemented mice showed no such changes. In addition, severe mitochondrial edema in the colon tissue of DSS-treated mice was observed according to TEM results. However, both low and high levels of supplementation alleviated these detrimental changes. Moreover, TUNEL staining indicated that DSS-induced mice had higher levels of apoptosis compared to both BA-supplemented mice and control mice.

### Effects of *B. amyloliquefaciens* on Immunoglobulins, Inflammatory Cytokines, and Colonic Infiltration Markers

**Figure 3:**
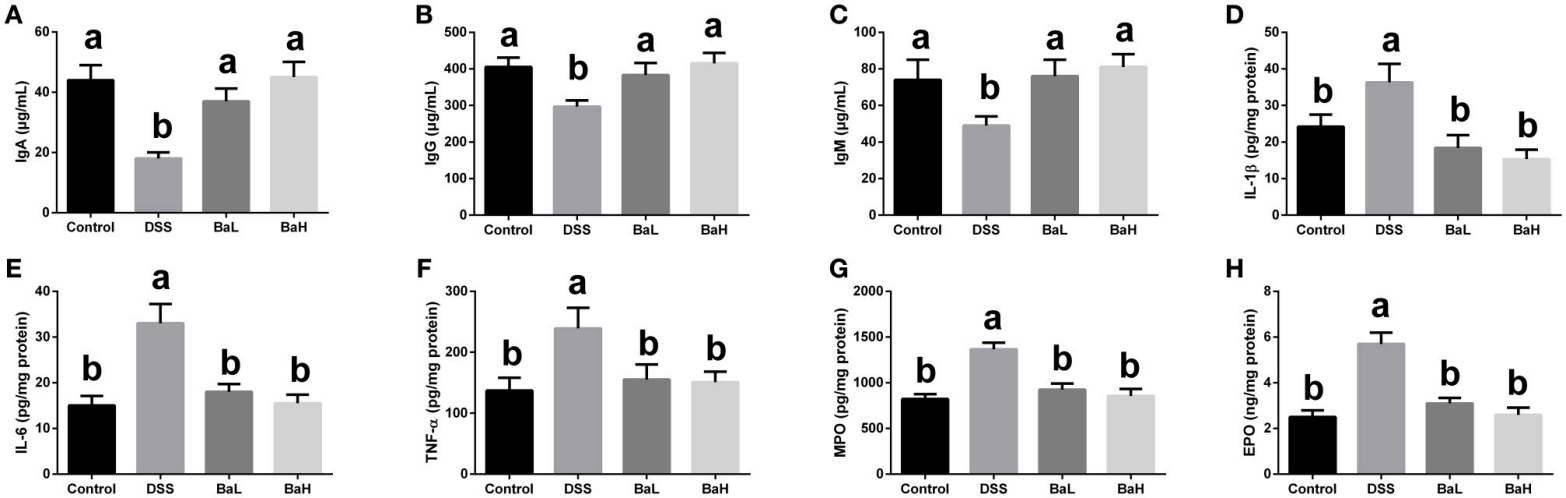
Effects of *B. amyloliquefaciens* on Immunoglobulins, Inflammatory Cytokines, and Colonic Infiltration Markers. **(A–C)** IgA, IgG, and IgM concentration in serum. **(D–F)** IL-1β, IL-6, and TNF-α concentration in colonic tissue. **(G,H)** MPO and EPO concentration in colonic tissue. Control, mice were untreated for 7 days and then orally gavaged PBS for 7 days; DSS, mice were supplemented with 3.5% DSS for 7 days and then orally gavaged PBS for 7 days; BaL, mice were supplemented with 3.5% DSS for 7 days and then orally gavaged *B. amyloliquefaciens* (Ba, 9.0 × 10^8^ CFU/kg in 200 μL of PBS/mouse/day) for 7 d; BaH, mice were supplemented with 3.5% DSS for 7 days and then orally gavaged *B. amyloliquefaciens* (Ba, 10.0 × 10^8^ CFU/kg in 200 μL of PBS/mouse/day). Values are expressed as mean ± SEM, *n* = 6. ^a,b^Means of the bars with different letters were significantly different among groups (*P* < 0.05).

IgA, IgG, and IgM concentrations (Figures 3A–C) in serum were decreased, whereas IL-1β, IL-6, and TNF-α concentrations in colonic tissue were increased in DSS-treated mice (Figures 3D–F) when compared with control mice. However, BA supplementation significantly alleviated these DSS-induced changes. In addition, MPO and EPO concentrations were increased in the colonic tissue of DSS-treated mice (Figures 3G,H) and those were significantly alleviated by supplementing DSS-treated mice with BA.

### Colonic Microbiota in Mice Is Altered After *B. amyloliquefaciens* Supplementation

**Figure 4:**
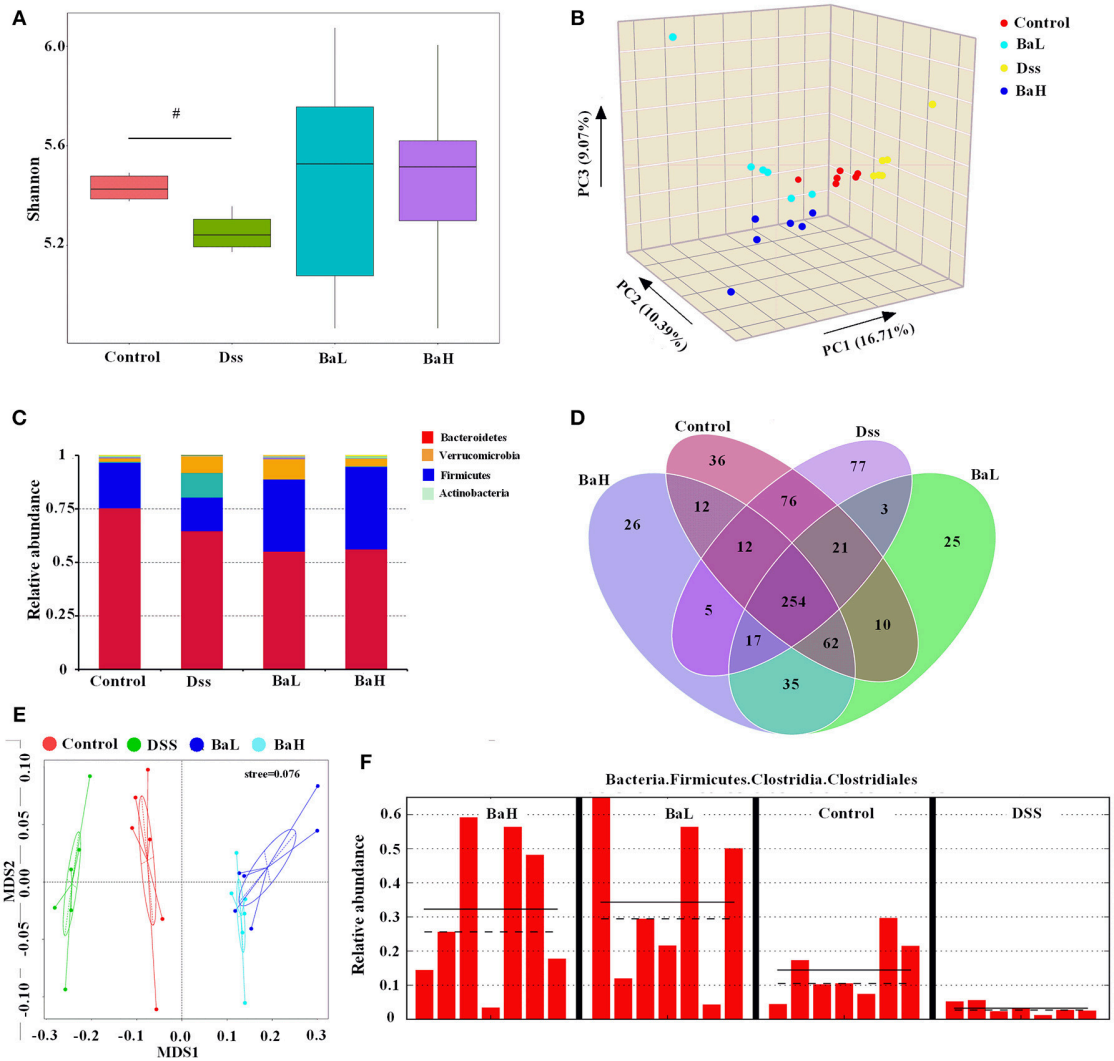
Colonic Microbiota in Mice is Altered after *B. Amyloliquefaciens* Supplementation. **(A)** Alpha diversity was estimated by the Shannon index. **(B)** PCoA plot of the microbiota based on an unweighted UniFrac metric. **(C)** Relative abundance of predominant bacteria at the phylum level. **(D)** Venn diagram of OTUs. **(E)** non-metric multidimensional scaling (NMDS) analysis. **(F)** Results of metagenomic biomarker discovery approach. Control, mice were untreated for 7 days and then orally gavaged PBS for 7 days; DSS, mice were supplemented with 3.5% DSS for 7 days and then orally gavaged PBS for 7 days; BaL, mice were supplemented with 3.5% DSS for 7 days and then orally gavaged *B. amyloliquefaciens* (Ba, 9.0 × 10^8^ CFU/kg in 200 μL of PBS/mouse/day) for 7 days; BaH, mice were supplemented with 3.5% DSS for 7 days and then orally gavaged *B. amyloliquefaciens* (Ba, 10.0 × 10^8^ CFU/kg in 200 μL of PBS/mouse/day). Values are expressed as mean ± SEM, *n* = 6. ^#^0.05 < *P* < 0.1 was considered as a tendency.

The hypervariable V3 + V4 regions of 16S rRNA genes were sequenced from colonic content and an average of 81,351 ± 15,327 reads were obtained. An average of 329 ± 61 operational taxonomic units (OTUs) were obtained after sequencing with at least 97% similarity. First, we determined the alpha diversity of colonic microflora and then investigated the differences in QIIME. Surprisingly, there was no significant alterations in alpha diversity as reflected by the observed species among all the groups, except that the Shannon index tend to change in DSS-treated mice (Figure 4A). However, a significant difference in beta-diversity among the four treatment groups were observed according to the weighted PCoA results (Figure 4B). To analyze the composition of bacterial communities, the top 10 phyla in terms of relative abundance among the colonic bacteria that presented in Control, DSS, BaL, and BaH groups were determined (Figure 4C). The dominant bacterial phyla in all samples collected from different mice were *Bacteroidetes, Firmicutes, Actinobacteria*, and *Verrucomicrobia*, which accounted for 98.2–99.7% of OTUs in mice. Remarkably, *Firmicutes* was decreased in DSS-treated mice when compared to that in mice of the other three groups, whereas no significant differences were observed among Control, BaL, and BaH groups. A Venn diagram, displaying the overlapping OTU data for all treatments, showed that 254 OTUs were shared among the colonic samples from all groups, based on this, the numbers of unique OTUs in Control, Dss, BaL, and BaH were 36, 77, 25, and 26, respectively (Figure 4D).

According to non-metric multidimensional scaling (NMDS) analysis, bacterial communities had similar structures in BaL and BaH groups, whereas DSS samples were extremely separated from the other three groups (Figure 4E). To fully understand the effects of BA treatments on DSS-induced mice, a metagenomic biomarker discovery approach (LEfSe) was used (Figure 4F). In the present study, taxa with an average relative abundance over >0.0001 in LEfSe to retain meaningful taxa. Only *Clostridiales* was observed to be significantly less abundant in the DSS group when compared to that in the Control group. Moreover, *Clostridiales* was significantly overrepresented in BaH and BaL groups when compared to its relative abundance in the Control group.

### Biofunction Predictions in Microbial Communities

**Figure 5:**
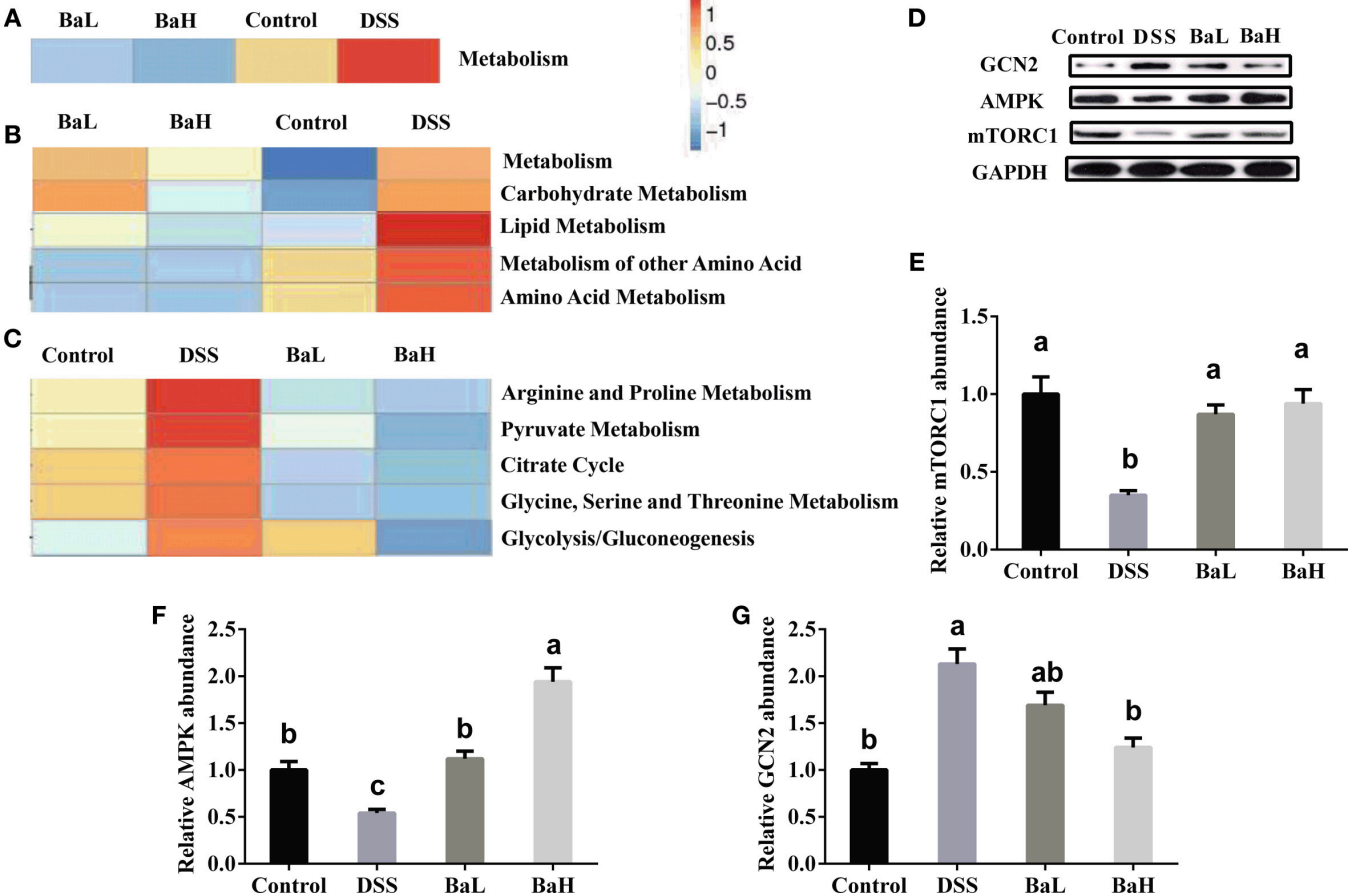
Biofunction Prediction of Microbial Communities and expression of proteins involved in metabolic signaling pathway in colonic tissue. KEEG pathway annotations in level 1 **(A)**, level 2 **(B)**, and level 3 **(C)**. Protein expression of mTORC1 **(D,E)**, AMPK **(D,F)** and GCN2 **(D,G)**. AMPK, AMP-activated protein kinase; GCN2, general amino acid control non-derepressible 2; mTORC1, mammalian target of rapamycin complex 1. Control, mice were untreated for 7 days and then orally gavaged PBS for 7 days; DSS, mice were supplemented with 3.5% DSS for 7 days and then orally gavaged PBS for 7 days; BaL, mice were supplemented with 3.5% DSS for 7 days and then orally gavaged *B. amyloliquefaciens* (Ba, 9.0 × 10^8^ CFU/kg in 200 μL of PBS/mouse/day) for 7 days; BaH, mice were supplemented with 3.5% DSS for 7 days and then orally gavaged *B. amyloliquefaciens* (Ba, 10.0 × 10^8^ CFU/kg in 200 μL of PBS/mouse/day). Values are expressed as mean ± SEM, *n* = 6. ^a,b,c^Means of the bars with different letters were significantly different among groups (*P* < 0.05).

To characterize the distinct functions of colonic microbiota, we performed PICRUSt analysis combined with the Kyoto Encyclopedia of Genes and Genomes (KEGG) database of microbial genomic information (Figure 5). This analysis permitted a comparison of the differences in the functional profiles among all groups and revealed pathways that were significantly different among Control, DSS, BaL, and BaH groups. The most significantly different KEGG pathway types were metabolism (predominantly amino acid, carbohydrate and lipid metabolism in level 2) pathways (Figures 5A,B). These pathways were further analyzed in KEGG level 3. The results showed that BA supplementation alleviated the increased activities of pyruvate, arginine, proline, glycine, serine, and threonine metabolism, as well as glycolysis/gluconeogenesis, and the citrate cycle, which were all induced by DSS treatment (Figure 5C). To further explore if metabolism in the small intestinal epithelium was changed in association with changes in the metabolic pathways of microbial communities, the protein expression of AMPK, GCN2, and mTORC1 was examined. We observed a significant increase in AMPK expression and a decrease in GCN2 and mTORC1 expression in BA-supplemented mice when compared to that in DSS-treated mice (Figures 5D–G).

## Discussion

*Bacillus amyloliquefaciens* have been widely used in animal feed as an alternative to antibiotics and no side effects were have previously been reported (Cao et al., 2018). In the present study, a strain of *B. amyloliquefaciens* (CGMCC 9384) was used to treat mice with DSS-induced colitis. The results showed that BA administration significantly ameliorated loss of body weight and colon weight, and decreased DAI in DSS-treated mice. Impaired intestinal structure and intestinal dysfunction such as mucosal and mitochondrial edema were associated with a disturbance in the balance of enterocyte apoptosis and renewal (Li et al., 2018). TUNEL staining showed higher rates of apoptotic cells and histological observation showed impaired intestinal villi in the colonic tissue of DSS-treated mice. However, we did not observe a difference between control mice and BA mice, which indicated the beneficial effects of BA treatment on intestinal morphology.

Impaired intestinal structure were also associated with immune activation and increased inflammatory response (Deniz et al., 2004; Xu et al., 2009). A previous study reported that the supplementation with BA, which was isolated and characterized from northeast Himalayan soil, decreased levels of pro-inflammatory cytokines in DSS-treated mice (Hairul Islam et al., 2011). Similarly, our data also showed that serum IgA, IgM, and IgG concentrations were also normalized by BA administration. In addition, infiltration of neutrophils and macrophages into the mucosa was also previously found to result in intestinal dysfunction related to colitis (Deniz et al., 2004). MPO and EPO activity are major factors that reflect granulocyte infiltration into colonic tissues (Han et al., 2015; Zhang et al., 2015). In the present study, the decreased levels of MPO and EPO in BA-administrated mice, when compared to those in DSS-treated mice, indicated the beneficial effects of alleviating inflammatory responses caused by the granulocytes.

For the new therapies to treat functional bowel disorders, dietary modifications focused on the gut microenvironment, mainly aimed at modulating the gut microbiota, have been studied most extensively (Simrén et al., 2013; Simrén and Tack, 2018). Trials also suggested that changes to the gut microbiota might play a role in the development of DSS-induced colitis (Nagalingam et al., 2011; Gkouskou et al., 2014; Jin et al., 2017). The administration of a mixture of probiotics including *Lactobacillus* and *Bifidobacterium* showed beneficial effects on colitis mouse models (Isaacs and Herfarth, 2008). Hence, dietary probiotics might alleviate colitis by modulating the intestinal microflora. In our present study, the Venn diagram showed the distinct OTUs appeared in the colonic microbiota in different groups. PCoA and NMDS analyses resulted in a clear separation between clusters of BA-treated and DSS-treated mice, indicating that BA administration transformed the colonic bacterial composition. Additionally, PCoA plots based on weighted UniFrac distance and the LEFSe analysis of pair-wise comparisons among BaL and BaH groups came to the agreement that there were no obvious differences between the different doses of BA used in the present study. From these perspectives, BA administration modulated the imbalanced colonic flora of DSS-treated mice. The *Bacteroidetes* to *Firmicutes* ratio is a reliable index to assess the composition of the gut microflora (Yelehe-Okouma et al., 2018). A shift to an increased *Firmicutes* to *Bacteroidetes* ratio was previously considered to be responsible for protection against inflammatory bowel disease (Cho and Blaser, 2012). Our present study found that *Firmicutes* was increased, whereas *Bacteroidetes* was decreased following BA administration, suggesting an increase in this ratio. These results indicated that BA might exert beneficial effects on the modulation of colonic microflora in DSS-treated mice.

PICRUSt analysis showed that metabolic pathways were most significantly differentially represented among all groups. Utilizing the KEGG database, marked increases in pathways of carbohydrate, lipid, and amino acid metabolism, as well as the metabolism of their secondary metabolites, were found in DSS-treated mice. Enhanced amino acid metabolism and the consumption of nutrients by microbial communities reduces the nutritional supply for intestinal cells. Meanwhile, the regeneration and proliferation of intestinal cells require a large amount of energy in the colon of DSS-treated mice. Hence, enterocyte renewal might be seriously affected in the colons of DSS-treated mice. However, this might not be a problem for mice administrated BA, as metabolism was not significantly different when compared to that in the control mice. GCN2 senses the absence of one or more amino acids, while the mTOR pathway could be activated by the presence of certain amino acids, such as leucine. In addition, AMPK, as the cellular energy sensor, plays a key role in regulating metabolism (Zhou et al., 2015). Consequently, the reduced expression of GCN2 and the enhanced expression of mTORC1 in the colonic tissue of mice administrated BA, as compared to levels in DSS-treated mice, suggested an increased supply of amino acids (Gallinetti et al., 2013). In addition, the expression of AMPK, the sensor of cellular energy, was increased, which indicated an increase in energy requirements for the enterocyte renewal.

In conclusion, our results showed that BA administration exerted beneficial effects on ameliorating weight loss, intestinal dysfunction, and inflammatory response during DSS-induced colitis in mice, suggesting that it could be useful for the treatment of dysbiosis during UC. Importantly, colonic microbiota composition in mice with colitis was significantly affected by BA administration. In addition, the biofunctional prediction of microbial communities indicated that changes of metabolism, and especially amino acid metabolism, might contribute to the beneficial effects of BA on the amelioration of DSS-induced colitis.

## Author Contributions

GCa and CY designed the trials, performed the experiments, and edited the manuscript. ZL and FT performed the samples detection. YX, JL, and GCh analyzed the data. GCa and CY wrote the manuscript, which was edited by ZL, FT, and KW. All authors read and approved the final manuscript.

### Conflict of Interest Statement

The authors declare that the research was conducted in the absence of any commercial or financial relationships that could be construed as a potential conflict of interest.
